# Association between Insulin Resistance and Breast Carcinoma: A Systematic Review and Meta-Analysis

**DOI:** 10.1371/journal.pone.0099317

**Published:** 2014-06-09

**Authors:** Adrian V. Hernandez, Mirella Guarnizo, Yony Miranda, Vinay Pasupuleti, Abhishek Deshpande, Socorro Paico, Hosten Lenti, Silvia Ganoza, Laritza Montalvo, Priyaleela Thota, Herbert Lazaro

**Affiliations:** 1 Instituto Médico de la Mujer/Instituto Médico Metabólico, Lima, Peru; 2 Postgraduate and Medical Schools, Universidad Peruana de Ciencias Aplicadas (UPC), Lima, Peru; 3 Health Outcomes and Clinical Epidemiology Section, Department of Quantitative Health Sciences, Lerner Research Institute, Cleveland Clinic, Cleveland, Ohio, United States of America; 4 Department of Medicine, Case Western Reserve University, Cleveland, Ohio, United States of America; 5 Department of Medicine, Medicine Institute, Cleveland Clinic, Cleveland, Ohio, United States of America; University of Eastern Finland, Finland

## Abstract

**Objective:**

This study was undertaken to evaluate the association between components defining insulin resistance and breast cancer in women.

**Study Design:**

We conducted a systematic review of four databases (PubMed-Medline, EMBASE, Web of Science, and Scopus) for observational studies evaluating components defining insulin resistance in women with and without breast cancer. A meta-analysis of the association between insulin resistance components and breast cancer was performed using random effects models.

**Results:**

Twenty-two studies (n = 33,405) were selected. Fasting insulin levels were not different between women with and without breast cancer (standardized mean difference, SMD −0.03, 95%CI −0.32 to 0.27; p = 0.9). Similarly, non-fasting/fasting C-peptide levels were not different between the two groups (mean difference, MD 0.07, −0.21 to 0.34; p = 0.6). Using individual odds ratios (ORs) adjusted at least for age, there was no higher risk of breast cancer when upper quartiles were compared with the lowest quartile (Q1) of fasting insulin levels (OR _Q2 vs. Q1_ 0.96, 0.71 to 1.28; OR _Q3 vs. Q1_ 1.22, 0.91 to 1.64; OR _Q4 vs. Q1_ 0.98, 0.70 to 1.38). Likewise, there were no differences for quartiles of non-fasting/fasting C-peptide levels (OR _Q2 vs. Q1_ 1.12, 0.91 to 1.37; OR _Q3 vs. Q1_ 1.20, 0.91 to 1.59; OR _Q4 vs. Q1_ 1.40, 1.03 to 1.92). Homeostatic model assessment (HOMA-IR) levels in breast cancer patients were significantly higher than in people without breast cancer (MD 0.22, 0.13 to 0.31, p<0.00001).

**Conclusions:**

Higher levels of fasting insulin or non-fasting/fasting C-peptide are not associated with breast cancer in women. HOMA-IR levels are slightly higher in women with breast cancer.

## Introduction

Breast cancer is the most common malignancy and the second leading cause of cancer death among women in the US. According to estimates for the year 2014, 235,030 breast cancer cases are expected to be newly diagnosed and 40,000 women will die from the disease in the US [1]. Breast cancer is a global health concern with worldwide estimates of more than one million women diagnosed with breast cancer every year, and more than 410,000 deaths from the disease, representing 14% of all female cancer deaths [2].

Insulin, a peptide hormone secreted by beta cells of the pancreas, promotes glucose absorption by cells and plays a central role in carbohydrate and fat metabolism. High insulin levels are a hallmark of insulin resistance. Insulin resistance is defined clinically as the inability of insulin to increase cellular glucose uptake and utilization, thereby leading to compensatory and chronic hyperinsulinemia [3].

Several epidemiologic studies have shown association between obesity and breast cancer in postmenopausal women [4], [5], [6]. Increased physical activity has been shown to decrease breast cancer risk in both pre and postmenopausal women [7]. Obesity and sedentary lifestyle are two significant predictors of development of insulin resistance and Type 2 diabetes mellitus (T2DM) [8]. The molecular mechanisms for these associations are unknown, but chronic sustained hyperinsulinemia in these insulin-resistant patients appears to play a role in the carcinogenesis. Several possible mechanisms have been proposed. Hyperinsulinemia amplifies bioavailablity of insulin like growth factor-1 (IGF-1), which together with insulin are known to promote human breast cancer [9]. Several studies have also shown an increase in breast cancer risk among women who have increased testosterone levels, reduced levels of sex hormone-binding globulin (SHBG), and hence elevated levels of bioavailable androgens and estrogens not bound to SHBG [10]. Collectively, these observations lead to the hypothesis that breast cancer risk may be increased in women with elevated plasma insulin levels.

Reliability of insulin and/or C-peptide levels as biomarkers of breast cancer has been a subject of controversy. Few studies report an association between these insulin resistance components and risk of breast cancer [11], [12] while other studies demonstrate a lack of an association [13], [14]. A recent meta-analysis of 6 prospective studies, found no evidence of an association between serum insulin or C-peptide concentrations and breast cancer risk [15]. Against this background, further investigation on this topic is warranted. Here we present a systematic review and meta-analysis of the association between components of insulin resistance and breast cancer.

## Materials and Methods

### Data sources and Searches

A comprehensive literature search using PubMed-Medline from 1960 through December 15, 2012, EMBASE from 1980 through December 15, 2012, The Web of Science from 1980 through December 15, 2012, and Scopus from 1960 through December 15, 2012 was conducted by three authors (AVH, VP and AD). The following keywords were used: hyperinsulinemia, breast cancer and breast carcinoma.

### Pubmed search strategy

(“hyperinsulinaemia”[All Fields] OR “hyperinsulinism”[MeSH Terms] OR “hyperinsulinism”[All Fields] OR “hyperinsulinemia”[All Fields]) AND ((“breast neoplasms”[MeSH Terms] OR (“breast”[All Fields] AND “neoplasms”[All Fields]) OR “breast neoplasms”[All Fields] OR (“breast”[All Fields] AND “cancer”[All Fields]) OR “breast cancer”[All Fields]) OR ((“breast”[MeSH Terms] OR “breast”[All Fields]) AND (“carcinoma”[MeSH Terms] OR “carcinoma”[All Fields])))

The following predetermined inclusion criteria was used: (i) observational studies evaluating the risk of components associated with insulin resistance and breast cancer, (ii) study population of patients ≥18 years; (iii) study in any language. Our exclusion criteria were: (i) no control group; (ii) fasting insulin, non-fasting/fasting c-peptide or HOMA-IR (glucose x insulin/a normalizing constant) data were not available or could not extracted for the study groups. Controls are defined as patients without breast cancer.

### Study selection and Data extraction

A list of retrieved articles was reviewed independently by 3 investigators (AVH, VP and AD) in order to choose potentially relevant articles, and disagreements about particular studies were discussed and resolved by consensus.

Two reviewers (VP and AD) independently extracted data from studies. The following information was extracted: age, gender, body mass index (BMI), menopausal status, insulin and/or c-peptide levels, method of diagnosis of breast cancer, fasting status when blood samples were collected, and assays for quantifying insulin and c-peptide. Information regarding homeostatic model assessment (HOMA-IR) scores was also collected, whenever available. One other author (AVH) reviewed the extractions for inconsistencies, and the three authors (AVH, VP and AD) reached consensus.

### Evaluation of Study Quality

The quality of the selected studies was assessed independently by two authors (V.P. and A.V.H.) using the Newcastle–Ottawa scale (NOS). The NOS uses two different tools for case–control and cohort studies and consists of three parameters of quality: selection, comparability and exposure/outcome assessment. The NOS assigns a maximum of four points for selection, two points for comparability and three points for exposure or outcome. NOS scores of ≥7 were considered as high quality studies and NOS scores of 5–6 were considered moderate quality. Any discrepancies were addressed by a joint re-evaluation of the original article.

### Data synthesis and analysis

Our systematic review and meta-analysis follow the recommendations of the Preferred Reporting Items for Systematic Reviews and Meta-Analyses (PRISMA) collaboration. DerSimonian and Laird random effects models were used for all meta-analyses [16]. We used the log odds ratios and their standard errors to combine ORs provided for specific tertiles or quartiles of continuous variables. We used random effects models to combine these log odds ratios, and then back transformed the association measures to provide ORs. When studies provided means of continuous outcomes (C-peptide and HOMA-IR), we used the mean difference to calculate summary statistics. However, when studies assessed the same outcome (fasting insulin) but measured in a variety of ways, we used standardized mean difference to calculate summary statistics. Standardized mean difference  =  difference in mean outcome between groups/standard deviation of outcome among participants. We evaluated statistical heterogeneity using the tau-squared (Tau^2^), Cochran Chi-square (χ^2^) and the I^2^ statistic [17], [18]. I^2^ values of 30–60% represented a moderate level of heterogeneity. A P value of <0.1 for χ^2^ was defined as indicating the presence of heterogeneity. Tau^2^ provides an estimate of between-study variance in random-effects meta-analysis. If Tau^2^ is >1, it suggests presence of substantial statistical heterogeneity. Publication bias was explored with the funnel plot and tested with the Egger's test of funnel plot asymmetry [19]. When the median and IQR were provided, the mean was estimated by the formula x =  (a+2m+b)/4 using the values of the median (m), P25 and P75 (a and b, respectively).We used Review Manager (RevMan, version 5.0 for Windows, Oxford, UK; The Cochrane Collaboration, 2008).

## Results

### Eligible studies

**Figure 1:**
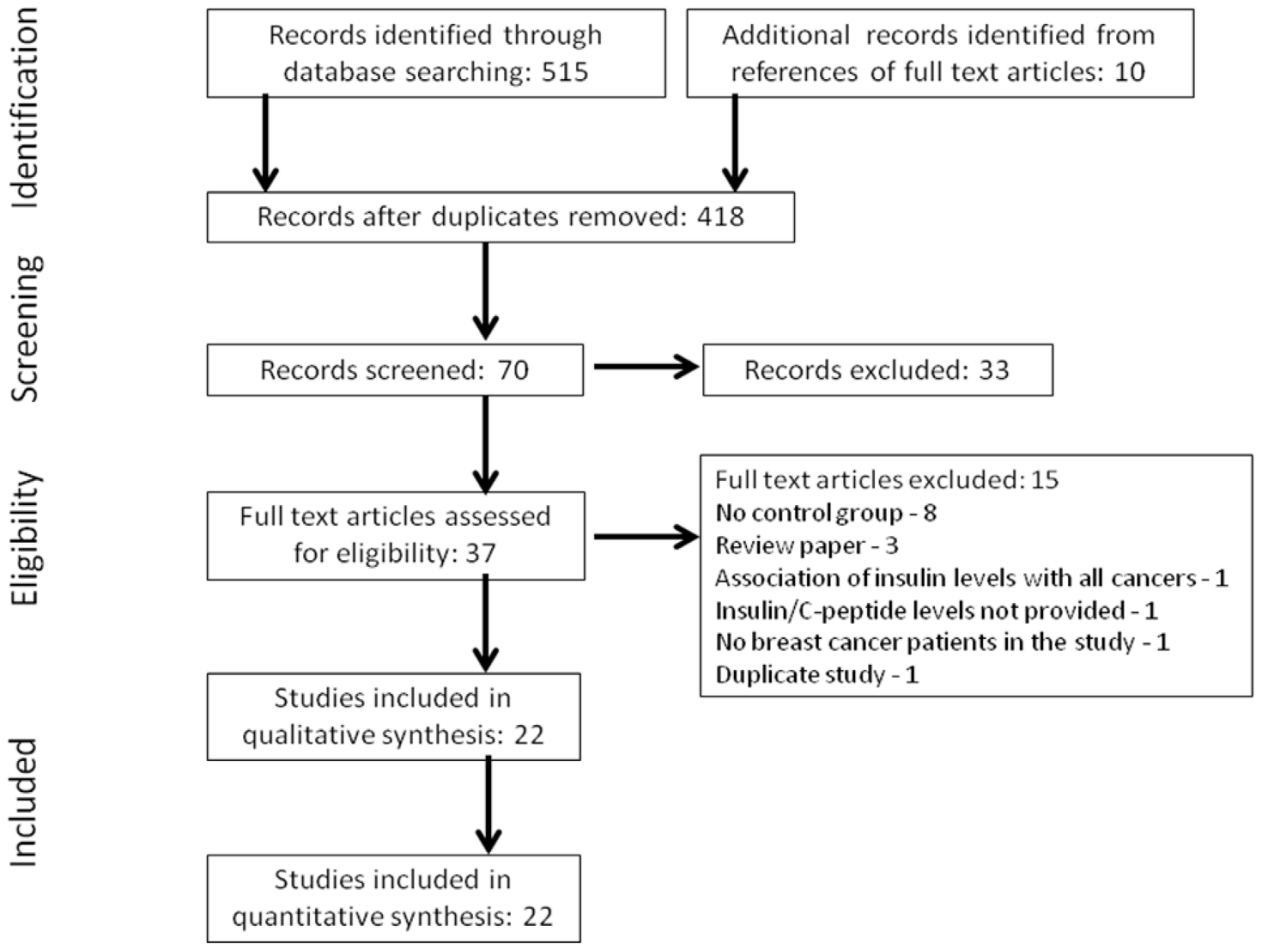
Flowchart of selected studies. Flow diagram showing the number of citations identified, excluded (with reasons for exclusion), and finally included in the meta-analysis.

Our search identified 525 publications (Figure 1). After removing duplicates, 418 articles were screened by title for relevance to study topic. Next, 70 articles were screened by abstract following which 37 articles were selected for full-text review based on relevance to the study topic and inclusion/exclusion criteria (Figure 1). Twenty-two studies that reported levels of components that define insulin resistance and their association with breast cancer in women were included in the meta-analysis. The reasons for exclusion of the remaining 15 articles are listed in Figure 1.

### Study characteristics

Table 1 summarizes the main characteristics of the included studies. Of the 22 studies included, 20 were case-control [11], [12], [13], [14], [20], [21], [22], [23], [24], [25], [26], [27], [28], [29], [30], [31], [32], [33], [34], [35] and 2 cross-sectional studies [36], [37]. Breast cancer cases were identified by pathology/medical records in 12 studies and cancer/tumor registries in 7 studies; no information was provided in 3 studies. Study population included premenopausal women only in 2 studies, postmenopausal women only in 7 studies and both pre- and post-menopausal women in 12 studies; no information was provided in one study. Only one study [23] provided information stratified for menopausal status. In studies providing data as tertiles/quartiles (fasting insulin, n = 7; non-fasting/fasting C-peptide, n = 8), the serologic variables were expressed as tertiles or quartiles based on their distributions in either controls or total study population. Eight studies provided the time lag data between measurement of insulin resistance and the diagnosis of breast cancer. Five studies provided median time interval (range: 2.2 yrs-6 yrs) whereas 3 studies provided mean time interval (range: 2.7 yrs-19 yrs). Of the 22 studies included in the meta-analysis the sample size ranged from 25 to 7,894. Breast cancer cases in the studies ranged from 2.4% to 53.8%.

**Table 1:** Patient characteristics in studies included in the meta-analysis.

Study reference, year	Year of publication	Country(ies)	Study design	Study Population	Sample size	Breast cancer cases (%)	Age, Mean (SD)	Blood samples	Biochemical assay	Breast cancer diagnosis	Time between measurement of IR and the diagnosis of cancer	Insulin resistance definition
Bruning PF *et al* ^20^	1992	Netherlands	CC	Pre and postmenopausal	664	33.6	NA	NonfastingC-peptide	Radioimmunoassay	NA	NA	NA
Yam D *et al* ^11^	1996	Israel	CC	NA	25	40.0	NA	Fasting insulin	Radioimmunoassay	Pathology	NA	NA
Del Giudice ME *et al* ^21^	1998	Canada	CC	Premenopausal	198	50.0	42.5 (5.1)	Fasting insulin	Radioimmunoassay	Pathology	NA	NA
Jernstrom H *et al* ^22^	1999	USA	CC	Postmenopausal	438	10.3	74.3 (9.5)	Fasting insulin	Radioimmunoassay	Pathology	NA	NA
Toniolo P *et al* ^23^	2000	USA	CC	Pre and postmenopausal	658	26.1	49.8 (8.4)	NonfastingC-peptide	Radioimmunoassay	Pathology records	Median: 57 months Range: 7–120 months	NA
Kaaks R *et al* ^24^	2002	Sweden	CC	Pre and postmenopausal	700	35.1	54.4	Fasting insulin	Immunoradiometric assay	Pathology	Median: 2.2 yrsRange: 1 month-10.6 yrs	NA
Mink PJ *et al* ^25^	2002	USA	CC	Pre and postmenopausal	7894	2.4	NA	Fasting insulin	Radioimmunoassay	Medical records and cancer registries	NA	NA
Keinan-Boker L *et al* ^26^	2003	Netherlands	CC	Postmenopausal	482	30.9	57.1 (5.5)	NonfastingC-peptide	Radioimmunoassay	Cancer registry	Median: 28 months Range: 14–73 months	NA
Lawlor DA *et al* ^36^	2004	UK	CS	Pre and postmenopausal	3837	3.8	68.9 (5.5)	Fasting insulin	ELISA assay	Medical records/database	Median: 6 yrs Range: 0.5–36 yrs	product of fasting glucose (mmol/l) and insulin (lU/ml) divided by the constant 22.5
Schairer C *et al* ^27^	2004	USA	CC	Postmenopausal	344	53.8	64.2 (9.6)	NonfastingC-peptide	Radioimmunoassay	Pathology	Mean: 19 yrs	NA
Gonullu G *et al* ^28^	2005	Turkey	CC	Postmenopausal	40	50.0	47.7 (4.0)	Fasting insulin	Radioimmunoassay	Pathology	NA	[(fasting glucose (mmol/L) x fasting insulin(mU/mL))/22.5]. Value greater than 2.7 was considered as insulin resistance; IR% = 37.5
Falk RT *et al* ^29^	2006	USA	CC	Premenopausal	357	47.8	38.6 (20-44)*	NonfastingC-peptide	Radioimmunoassay	Pathology	NA	NA
Verheus M *et al* ^30^	2006	8 european countries	CC	Pre and postmenopausal	3345	34.1	54.5	Fasting and nonfasting C-peptide	Radioimmunoassay	Cancer and pathology registries	Mean: 2.8 yrs Range: 0.1–6.3 yrs	NA
Eliassen AH *et al* ^14^	2007	USA	CC	Pre and postmenopausal	617 (insulin)	33.7	45.0	Fasting insulin	Radioimmunoassay	Medical records	Mean: 31 months Range: 1–87 months	NA
					945 (c-peptide)	33.4		Fasting and nonfasting C-peptide	ELISA assay			
Fair AM *et al* ^12^	2007	China	CC	Pre and postmenopausal	794	50.0	47.7 (7.9)	C-peptide (fasting status NA)	ELISA assay	Cancer registry	NA	NA
Garmendia ML *et al* ^13^	2007	Chile	CC	Pre and postmenopausal	340	50.0	55.8 (11.4)	Fasting insulin	ELISA assay	Pathology	NA	Insulin resistance was measured by HOMA = insulin/(22.5e^-lnglucose^) and defined as 2.5 or more
												IR% = 55.3
Cust AE *et al* ^31^	2009	Sweden	CC	Pre and postmenopausal	1122	50.0	NA	NonfastingC-peptide	Radioimmunoassay	Cancer registry	Median: 4.4 yrs	NA
Gunter MJ *et al* ^32^	2009	USA	CC	Postmenopausal	1651	50.9	63.5	Fasting insulin	NA	Medical records and tumor registries	NA	HOMA-IR index = fasting insulin [µ IU/mL] × fasting glucose [mg/dL]/22.5
Kabat GC *et al* ^33^	2009	USA	CC	Postmenopausal	5450	3.5	62.6 (6.6)	Fasting insulin	ELISA assay	Medical records and tumor registries	NA	([fasting insulin(microIU/mL) x fasting glucose (mg/dL)]/22.5)
Abbasi M *et al* ^37^	2010	Iran	CS	Pre and postmenopausal	920	8.9	38.1 (12.0)	Fasting insulin	Radioimmunoassay	Pathology	NA	fasting insulin (mU/L) x fasting glucose (mg/dL)/405. HOMA-IR values of greater than 1.8 as indicative for insulin resistance
Capasso I *et al* ^34^	2011	Italy	CC	Postmenopausal	777	37.7	57.55 (45–75)*	Insulin (fasting status NA)	NA	NA	NA	NA
Sieri S *et al* ^35^	2012	Italy	CC	Pre and postmenopausal	1807	20.6	NA	Fasting insulin	Chemiluminescent immunoassay	NA	NA	Not defined

NA  =  not available; CS  =  cross-sectional; CC  =  case-control; *  =  Mean (range); HOMA-IR  =  Homeostatic model of assessment-insulin resistance; IR  =  Insulin resistance.

### Quality Assessment

Using the NOS scale, all but 2 studies [11], [34] were identified as high quality (reported in Table S1 in File S1). All studies clearly identified the study population and defined the outcome and outcome assessment (reported in Table S2 in File S1). All but 2 studies [11], [34] identified important confounders or prognostic factors and were used for adjustment of the association between insulin/c-peptide levels and breast cancer. There was considerable variation in the selection of confounding variables for adjustment (reported in Table S2 in File S1). It is possible that a few confounding variables were not fully identified and recorded. The most common confounder adjusted was age.

### Publication bias

The funnel plot did not suggest the presence of publication bias, and the formal test of asymmetry of this plot was not significant (Egger's p value = 0.8).

### Meta-analyses

**Figure 2:**
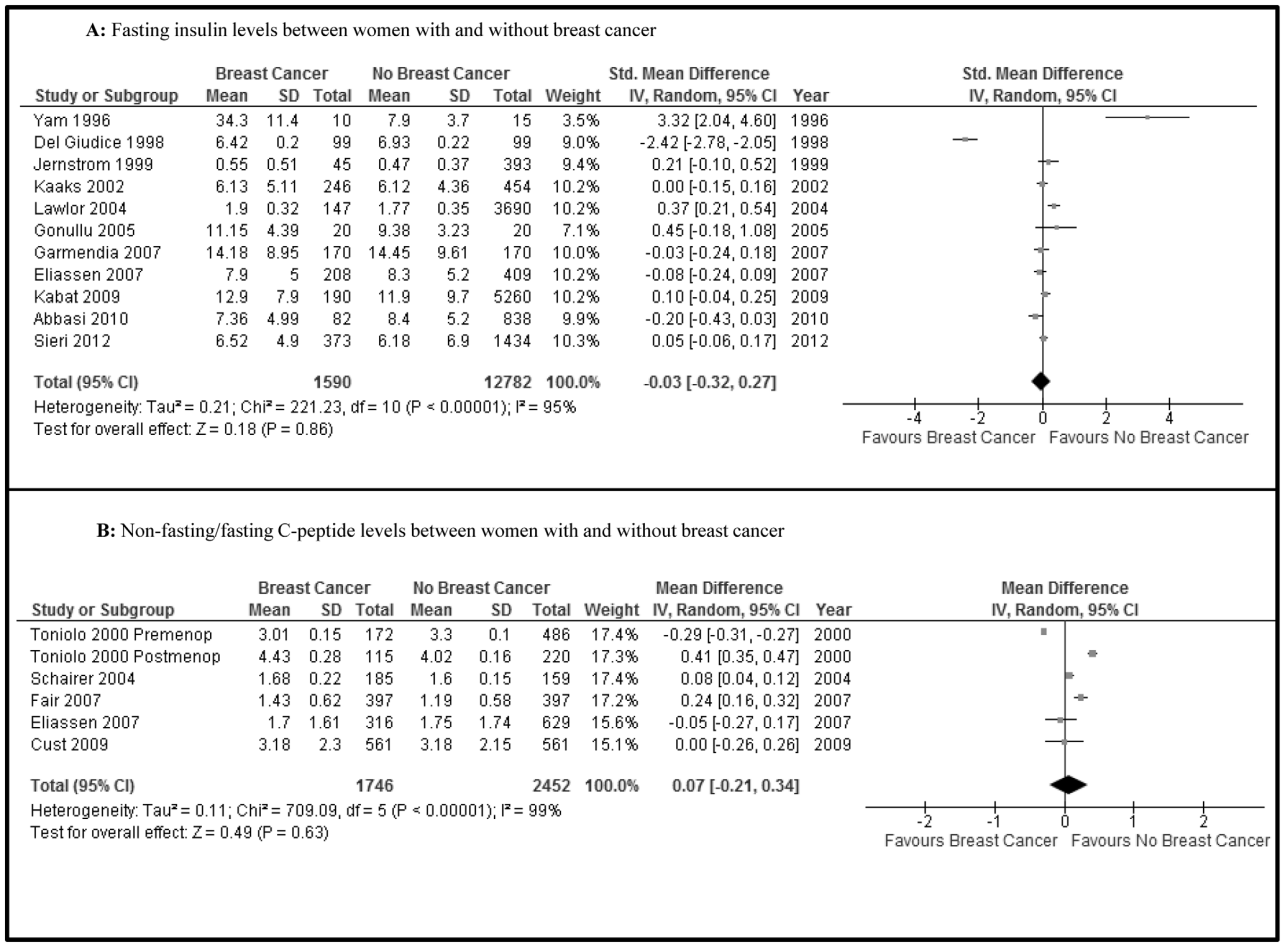
Forest plot of observational studies comparing women with and without breast cancer for (A) Fasting insulin levels and (B) Non-fasting/fasting C-peptide levels. (IV, Random  =  Inverse variance, Random effects model.)

**Figure 3:**
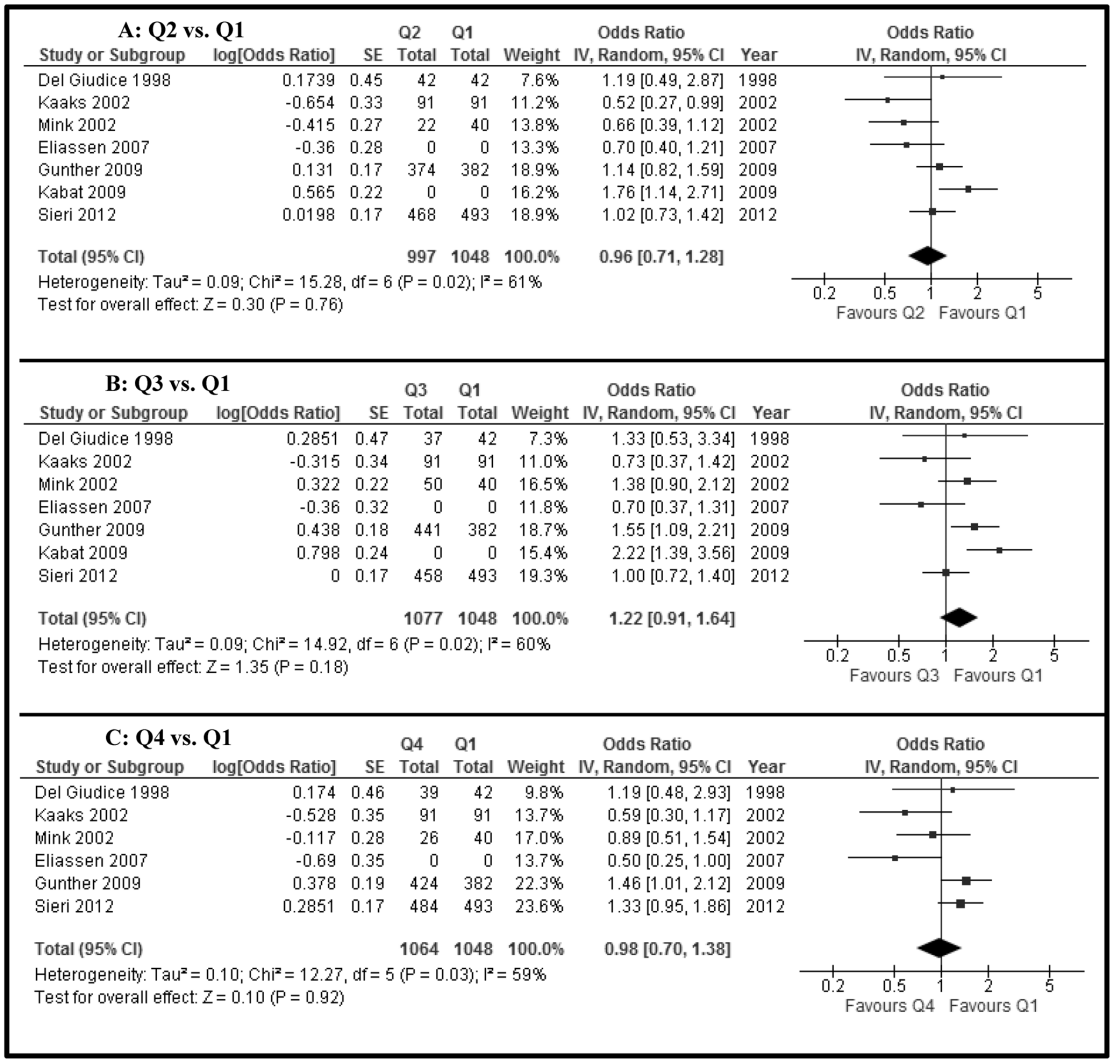
Forest plot of observational studies with adjusted ORs for breast cancer between quartiles of fasting insulin levels (A) Q2 versus Q1; (B) Q3 versus Q1; (C) Q4 versus Q1 ORs. (IV, Random =  Inverse variance, Random effects model.)

**Figure 4:**
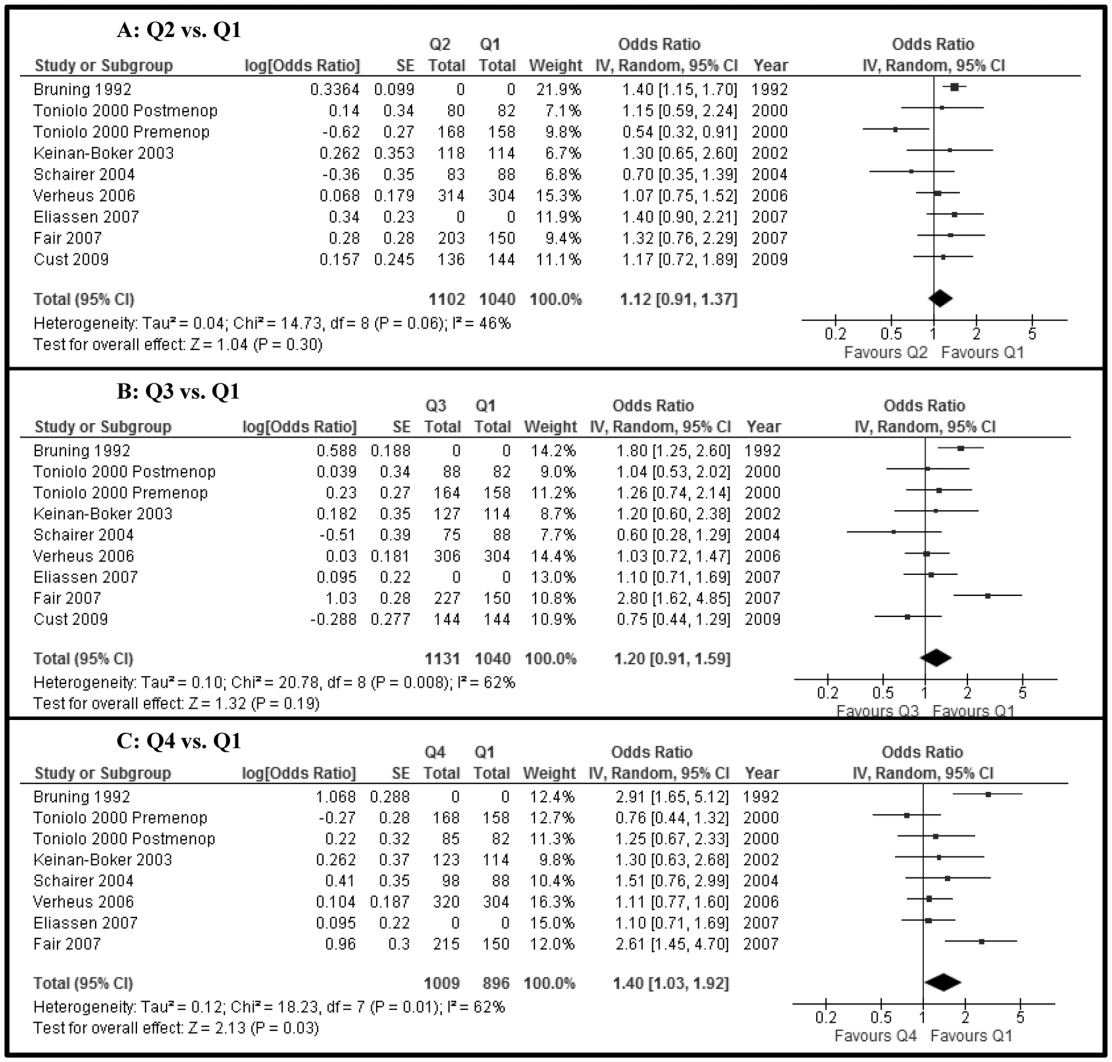
Forest plot of observational studies with adjusted ORs for breast cancer between quartiles of non-fasting/fasting C-peptide levels (A) Q2 versus Q1; (B) Q3 versus Q1; (C) Q4 versus Q1. (IV, Random =  Inverse variance, Random effects model.)

**Figure 5:**
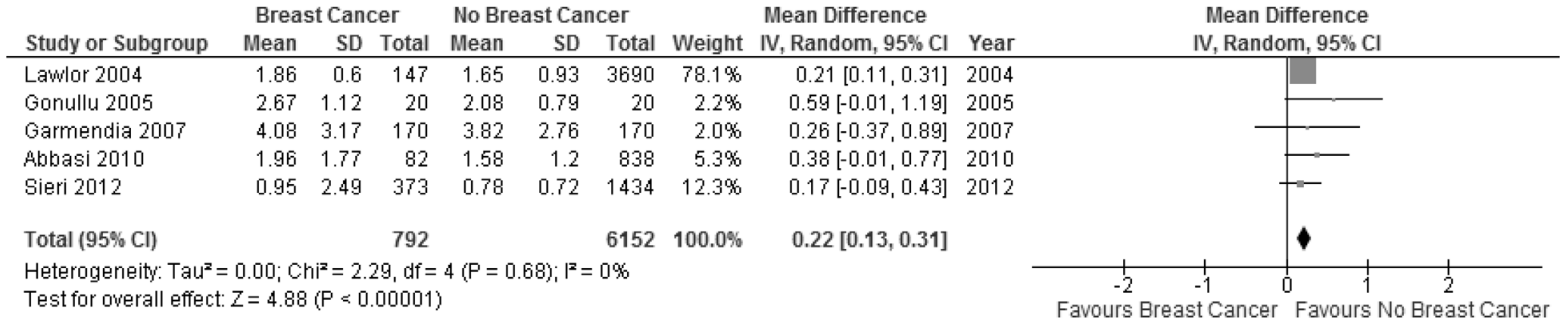
Forest plot of observational studies comparing women with and without breast cancer for HOMA-IR levels. (IV, Random  =  Inverse variance, Random effects model.)

The fasting insulin levels (11 studies, n = 14,372) were not different between women with and without breast cancer (SMD −0.03, 95% CI −0.32 to 0.27, P = 0.9) (Figure 2A). Furthermore, non-fasting/fasting C-peptide levels (5 studies, n = 4,198) were not different between the two groups (MD 0.07, −0.21 to 0.34, p = 0.6) (Figure 2B). There was high heterogeneity (I^2^ = 95% and 99%, respectively) for these two associations. Using individual ORs adjusted for age at least, there was no increased risk of breast cancer when the higher quartiles were compared with the lowest quartile (Q1) of fasting insulin (Q2 vs Q1, 7 studies, n = 2,045; Q3 vs Q1, 7 studies, n = 2,125; Q4 vs Q1, 6 studies, n = 2,112) (OR _Q2 vs Q1_ 0.96, 0.71 to 1.28; OR _Q3 vs Q1_ 1.22, 0.91 to 1.64; OR _Q4 vs. Q1_ 0.98, 0.70 to 1.38) (Figures 3A, 3B and 3C, respectively). Also, there were no differences for quartiles of non-fasting/fasting C-peptide levels (Q2 vs Q1, 8 studies, n = 2,142; Q3 vs Q1, 8 studies, n = 2,171; Q4 vs Q1, 7 studies, n = 1,905) (OR _Q2 vs Q1_ 1.12, 0.91 to 1.37; OR _Q3 vs Q1_ 1.20, 0.91 to 1.59; OR _Q4 vs. Q1_ 1.40, 1.03 to 1.92) (Figures 4A, 4B and 4C, respectively). The level of heterogeneity on pooling ORs adjusted for age at least was moderate. HOMA-IR levels (5 studies, n = 6,944) in women with breast cancer were significantly and slightly higher than in women without breast cancer (MD 0.22, 0.13 to 0.31, p<0.00001; I^2^ = 0) (Figure 5).

## Discussion

In this systematic review we did not find differences between fasting insulin or non-fasting/fasting C-peptide levels and women with and without breast cancer. There was no higher adjusted risk of breast cancer between higher quartiles of fasting insulin or non-fasting/fasting C-peptide levels and their lowest quartile levels. Finally, fasting HOMA-IR levels were slightly and significantly higher in women with breast cancer in comparison with women without breast cancer.

Insulin and IGF-1 are peptide hormones that stimulate proliferation of tissues and levels higher than normal (i.e. hyperinsulinemia) have been linked with carcinogenic properties in animal models [38]. Hyperinsulinemia occurs in presence of insulin resistance, a key pathophysiological mechanism linked to type 2 diabetes mellitus and obesity. There is substantial epidemiological evidence linking insulin resistance and cancer of the liver, colon and pancreas [39]. However, the association between hyperinsulinemia/insulin resistance and breast cancer has been controversial in humans. Several other mechanisms apart from hyperinsulinemia have been described for breast cancer such as increased angiogenesis, hypoadiponectinemia, and increased bioactivity of estrogens and testosterone [40].

A meta-analysis has linked diabetes mellitus and breast cancer risk [41]. These investigators studied 20 observational studies of diabetic patients with 39,719 cases of breast cancer. There was a modest association between diabetes and breast cancer (OR 1.15, 95% CI 1.12–1.19), which was higher for postmenopausal women than for premenopausal women (OR 1.19, 95%CI 1.15–1.23 and OR 0.94, 95% CI 0.80–1.10, respectively). A high level of heterogeneity was observed among association effects. Obesity and hyperinsulinemia were described as the main potential mechanisms of this positive association.

Another recent meta-analysis evaluated the relationship between metabolic syndrome and breast cancer [42]. Metabolic syndrome as defined by the WHO includes insulin resistance amongst the diagnostic criteria, along with two or more of the following: increased waist-to-hip ratio, hypertriglyceridemia, low HDL cholesterol, high blood pressure and microalbuminuria [43]. Eleven studies involving 9,643 breast cancer cases were analyzed and a weak association between metabolic syndrome and breast cancer was found (RR 1.23, 95% CI 1.05–1.45). Within postmenopausal studies (5 studies, 1,290 breast cancer cases) there was a slightly higher association was found: RR 1.56, 95% CI 1.08–2.24. Importantly, also a substantial heterogeneity of association effects was observed among studies. Other diagnostic criteria of metabolic syndrome such as waist-to-hip ratio, hypertension, and low HDL cholesterol have not individually shown consistent associations with breast cancer [41].

We did not find any association between markers of insulin resistance and breast cancer. Fasting insulin levels and non-fasting/fasting C-peptide levels may shadow the state of stress that the pancreas has in presence of insulin resistance. We believe that measuring insulin levels in the 3-hour period after a sugar load test can provide better information and potentially peak levels of insulin or the area below the 3-hour insulin curve may be associated with breast cancer risk. Higher fasting insulin levels have been slightly associated with higher risk of endometrial cancer (OR highest quartile vs. lowest quartile of insulin 1.64, 95%CI 1.12–2.40) [44].

Load tests may give higher chances to find an association not found with current measurements. Previous research has shown an association between glycemic load and breast cancer risk in a meta-analysis [45]. The glycemic load combines the loads for the total servings of all carbohydrate-containing foods consumed per day, on average. Glycemic load has been associated with a slightly higher risk of breast cancer (RR 1.14, 95% CI 1.02–1.28). Similarly, the association of glycemic load with endometrial cancer was weak (RR 1.36, 95% CI 1.14–1.62).

We did not evaluate fasting glucose individually, but we did evaluate HOMA-IR scores, which is proportional to glucose x insulin levels. A very small, although significant association between HOMA-IR scores and breast cancer was found. Unfortunately, we could not explore the association between higher quartiles and the lowest quartile of HOMA-IR scores, as they were not available in the selected studies. Again, we propose that glucose and insulin levels may be both measured after a sugar load test and these values may provide a better estimate of the association of insulin resistance and breast cancer risk.

A recent meta-analysis found no evidence of an association between serum insulin and C-peptide concentration and breast cancer in 6 prospective studies with 1,890 cases [15]. We conducted a comprehensive search and evaluated this association in all observational studies to date. Our meta-analysis included 22 studies with 7,478 cases. We also determined the association between HOMA-IR scores and breast cancer. In our meta-analysis studies investigating the associations between insulin/C-peptide levels and breast cancer were heterogenous as was the case with the meta-analysis of prospective studies only.

Our study has several limitations. First, the observational nature of the included studies may weaken our conclusions, especially because most are case-control studies. However, the risk of bias of included studies was low. Second, although we compared insulin, C-peptide levels and HOMA-IR scores univariably between women with and without breast cancer, we also meta-analyzed risks measures of breast cancer between quartiles that were adjusted at least for age, and in most of cases for several essential confounders. Third, there was substantial clinical and statistical heterogeneity among included studies. Previous meta-analyses [41], [42] investigating factors associated with breast cancer also showed high levels of heterogeneity; we could not perform analyses by age subgroups because data was not provided as such, and by menopausal status as only one of the studies provided that information.

In observational studies, higher levels of fasting insulin or non-fasting/fasting C-peptide were not found to be associated with the presence of breast cancer in women, even after adjustment for important confounders. HOMA-IR levels are slightly higher in women with breast cancer.

## Supporting Information

Checklist S1 **PRISMA Checklist.**
(DOC)

File S1 **Supporting tables. Table S1, Newcastle-Ottawa Scale. Table S2, Study Quality Assessment.**
(DOC)
