# Supplementary material for: Association between Insulin Resistance and Breast Carcinoma: A Systematic Review and Meta-Analysis
Source: PLoS One. 2014 Jun 9;9(6):e99317. doi: 10.1371/journal.pone.0099317 (PMC4049776; doi:10.1371/journal.pone.0099317)
Supplement: File S1 — Supporting tables. Table S1, Newcastle-Ottawa Scale. Table S2, Study Quality Assessment. (DOC) [file pone.0099317.s002.doc]

| **Table S1: Newcastle-Ottawa Scale** | | | | | | | | | | |
| --- | --- | --- | --- | --- | --- | --- | --- | --- | --- | --- |
| **First author** | **Case definition**  **adequate?** | **Representativeness**  **of the cases** | **Selection**  **of controls** | **Definition**  **of controls** | **Comparability of cases and controls** | | **Ascertainment**  **of exposure** | **Same method of ascertainment**  **for cases and controls** | **Non-response**  **rate** | **Total**  **score** |
|  |  |  |  |  | **controls for age** | **controls for any additional factor** |  |  |  |  |
| Bruning PF *et al* | 1 | 1 | 1 | 0 | 1 | 1 | 1 | 1 | 1 | 8 |
| Yam D *et al* | 1 | 0 | 0 | 0 | 0 | 0 | 1 | 1 | 1 | 4 |
| Del Giudice ME *et al* | 1 | 0 | 0 | 1 | 1 | 1 | 1 | 1 | 1 | 7 |
| Jernstrom H *et al* | 1 | 1 | 1 | 1 | 1 | 1 | 1 | 1 | 1 | 9 |
| Toniolo P *et al* | 1 | 1 | 1 | 0 | 1 | 1 | 1 | 1 | 1 | 8 |
| Kaaks R *et al* | 1 | 1 | 1 | 1 | 1 | 1 | 1 | 1 | 0 | 8 |
| Mink PJ *et al* | 1 | 1 | 1 | 0 | 1 | 1 | 1 | 1 | 0 | 7 |
| Keinan-Boker L *et al* | 1 | 1 | 1 | 1 | 1 | 1 | 1 | 1 | 1 | 9 |
| Lawlor DA *et al* | 1 | 1 | 1 | 1 | 1 | 1 | 1 | 1 | 1 | 9 |
| Schairer C *et al* | 1 | 1 | 1 | 0 | 1 | 1 | 1 | 1 | 1 | 8 |
| Gonullu G *et al* | 1 | 1 | 1 | 0 | 0 | 1 | 1 | 1 | 1 | 7 |
| Falk RT *et al* | 1 | 1 | 1 | 0 | 1 | 1 | 1 | 1 | 0 | 7 |
| Verheus M *et al* | 1 | 1 | 1 | 1 | 1 | 1 | 1 | 1 | 1 | 9 |
| Eliassen AH *et al* | 1 | 1 | 1 | 1 | 1 | 1 | 1 | 1 | 1 | 9 |
| Fair AM *et al* | 1 | 1 | 1 | 1 | 1 | 1 | 1 | 1 | 1 | 9 |
| Garmendia ML *et al* | 1 | 1 | 1 | 1 | 1 | 1 | 1 | 1 | 0 | 8 |
| Cust AE *et al* | 1 | 1 | 1 | 1 | 1 | 1 | 1 | 1 | 1 | 9 |
| Gunter MJ *et al* | 1 | 1 | 1 | 1 | 1 | 1 | 1 | 1 | 0 | 8 |
| Kabat GC *et al* | 1 | 1 | 1 | 0 | 1 | 1 | 1 | 1 | 1 | 8 |
| Abbasi M *et al* | 1 | 1 | 1 | 0 | 1 | 1 | 1 | 1 | 1 | 8 |
| Capasso I et al | 1 | 1 | 1 | 0 | 0 | 0 | 1 | 1 | 1 | 6 |
| Sieri S *et al* | 1 | 1 | 1 | 1 | 1 | 1 | 1 | 1 | 0 | 8 |

| **Table S2: Study Quality Assessment** | | | | |
| --- | --- | --- | --- | --- |
| **Study** | **Study population clearly identified?** | **Clear definition of outcome and outcome assessment?** | **Important confounders and/ prognostic factors identified?** | **Newcastle-Ottawa Scale scores** |
| Bruning PF *et al* | Yes | Yes | Adjusted for age, menopausal status, family history of breast cancer, BMI, and WHR | 8 |
| Yam D *et al* | Yes | Yes | None | 4 |
| Del Giudice ME *et al* | Yes | Yes | Adjusted for age and weight | 7 |
| Jernstrom H *et al* | Yes | Yes | Adjusted for age, age at menopause, age at menarche, weight and no. of live births | 9 |
| Toniolo P *et al* | Yes | Yes | Adjusted for age, menopausal status, date of blood sampling, phase of menstrual cycle, history of benign breast disease and parity | 8 |
| Kaaks R *et al* | Yes | Yes | Adjusted for age, menopausal status and use of pill/exogenous hormones | 8 |
| Mink PJ *et al* | Yes | Yes | Adjusted for age, race, BMI, study center, age at menopause, age at menarche, age at first livebirth, family history of breast cancer, number of sisters, alcohol intake and pack-years of smoking | 7 |
| Keinan-Boker L *et al* | Yes | Yes | Adjusted for age, BMI, residency, date of enrollment, age at menarche, age at first fullterm delivery and IGFBP-3 levels | 9 |
| Lawlor DA *et al* | Yes | Yes | Adjusted for age, age at menopause/menarche, parity, oral contraception use, social class, cigarette smoking, HRT use, BMI and WHR | 9 |
| Schairer C *et al* | Yes | Yes | Adjusted for age at diagnosis, age at menopause, Quetelet index, nulliparity, year of diagnosis, time of blood draw, IGF-1, IGFBP-3 and fructosamine | 8 |
| Gonullu G *et al* | Yes | Yes | Adjusted for BMI | 7 |
| Falk RT *et al* | Yes | Yes | Adjusted for age, BMI, time of blood collection and phase of menstrual cycle | 7 |
| Verheus M *et al* | Yes | Yes | Adjusted for age, study center, menopausal status, time of sample collection, fasting status, phase of menstrual cycle and free testosterone/estradiol | 9 |
| Eliassen AH *et al* | Yes | Yes | Adjusted for age, menopausal status, month/year of blood draw, race/ethnicity, fasting status, BMI, age at menarche, parity, family history of breast cancer, and history of benign breast disease | 9 |
| Fair AM *et al* | Yes | Yes | Adjusted for age, menopausal status and date of sample collection | 9 |
| Garmendia ML *et al* | Yes | Yes | Adjusted for age and neighborhood of residence | 8 |
| Cust AE *et al* | Yes | Yes | Adjusted for age, date of blood sampling and fasting status | 9 |
| Gunter MJ *et al* | Yes | Yes | Adjusted for age, BMI, race, alcohol consumption, smoking, family history of breast cancer, parity, age at menopause, age at menarche, age at first child’s birth, use of OCPs, use of NSAIDs, HRT use, use of estrogen replacement therapy, educational attainment and physical activity | 8 |
| Kabat GC *et al* | Yes | Yes | Adjusted for age, ethnicity, education, BMI, waist circumference, OCP use, HRT, age at menarche, age at menopause, age at first birth, alcohol, total energy intake, family history of breast cancer, history of breast biopsy, DM, physical activity, randomization status in hormone therapy, calcium plus vit. D and dietary modification trials. | 8 |
| Abbasi M *et al* | Yes | Yes | Adjusted for age and central obesity | 8 |
| Capasso I *et al* | Yes | Yes | None | 6 |
| Sieri S *et al* | Yes | Yes | Adjusted for age, menopausal status, length of storage of serum samples, education, age at first birth, age at menarche, parity, family history of breast cancer, oral contraception use, breastfeeding, alcohol intake and smoking | 8 |
| BMI = Body mass index; HRT = Hormone replacement therapy; WHR = Waist to hip ratio; IGF-1 = Insulin-like growth factor-1; IGFBP-3 = Insulin-like growth factor binding protein-3; OCP = oral contraceptive pills; NSAID; Non-steroidal anti-inflammatory drugs; DM = diabetes mellitus | | | | |
